# The Treatment of Putrescent Teeth

**Published:** 1907-07-15

**Authors:** L. P. Hall


### THE TREATMENT OF PUTRESCENT TEETH.

BY L. P. HALL, D. D. S.

Read before the Detroit Dental Society, April 11, 1907.

The treatment of teeth becomes a burden to us, or affords us great satisfaction, according to our success or failure.

In the treatment of putrescent pulps, one of the essentials of success is a patient who will present himself for treatment when expected, in order that we may push the treatment as rapidly as possible, and that we may note any changes that are taking place.

I have no use for a patient who fails to keep an appointment because of some trivial cause, and I do not hesitate to let him know how I feel about it.

Upon our part, we must do more than open the mouth and hurriedly change a little cotton dressing, too often pushing it through saliva and debris, and dismiss the patient. We must have a thorough knowledge of the anatomy of the tooth to be treated. We must be methodical, studying each case sufficiently to diagnose it properly. And above all we must be careful to treat it antiseptically.

I trust you will bear with me for going so minutely into the instrumentation in the preparation and treatment of a tooth.

In every case where possible, the dam should be placed on the tooth treated, and upon such adjacent teeth as will render that tooth more accessible and free the operation from possible contamination.

Teeth become putrescent through septic matter sealed under filings, or by septic matter being carried through the blood supply to the apex of a tooth that has long been dead from some traumatic cause, and which now affords a fertile culture medium.

The most common cause of putrescent pulp is infection through an exposure in a cavity, but after the pulp dies it may give no trouble until the cavity or orifice becomes clogged with food or debris. In these cases it is well first to remove the stoppage, and thereby remove the gas pressure. Flush out with warm sterile water all the debris before placing the dam. After the dam is placed, dry out the cavity, wash with alcohol and dry with absorbents and hot air. If the tooth is not too sore, cut back all over-hanging walls for good access, and remove carefully all decay from cavity, making it as clean as possible at first sitting. If the canals are not clearly defined, cut out over the floor of the cavity with a large round burr; removing all discolored dentine. If the canals, or any of them, are not readily seen, do not hunt them up with a small burr. For this purpose use a very fine, smooth, steel probe. I keep several of these, which I never use for anything else.

Having located the canals, tease out from them with *fine* broaches the remnants of pulp tissue, being careful not to force anything through the apical foramen. A fine broach is suggested, because if the broach is nearly the size of the canal, it will tend to act as a piston and force the contents ahead of it and through the foramen. Flood the cavity and canals occasionally with alcohol, and dry with hot air again, in order to sterilize as far into the canals as possible each time. Alcohol is used here because it is easily dried, and with the parts dry you are less likely to force any debris through.

When a portion of the contents of the canals has been removed, the orifices of the canals (if very irregular or small), can then be enlarged or funneled out with a bud-shaped bur to permit of easy entrance. This is of great importance in those cases that are just out of the line of vision, because in the supsequent treatments the moment the broach or probe reaches this larger bevel, it more readily enters the canal without the aid of a mirror each time.

After again flushing with alcohol and drying the the canals, spiral broaches may be used. These, because of their form, tend to withdraw the contents of the canal as an auger draws out the wood in cutting, rather than force it in.

If it now appears that the apical third of the nerve is still alive, the canal should be dried and a very little arsenic sealed in, or, if you feel sure all is sterile, a very little cocaine can be applied with pressure. Be careful not to use much pressure, a very little will suffice.

Remove the filaments, flood the canals with eucalyptol or iodized phenol, and seal the cavity for a day or two, painting the gums about the tooth with iodine and aconite after removal of the dam.

If there is no life in the pulp at all, or the case has progressed so far that there is more or less pus about the apex of the root, as is indicated by a considerable flow of pus upon opening the tooth, the parts on either side of the root should be gently pressed with the thumb and fingers to express as much pus as possible. Often the patient will be able to do this pressing much more effectively than the operator, and stand it with a better grace. If a little hemorrhage begins to show, it is an indication that the pus is about out. Then dry dressings should be introduced until all moisture is removed, and canal seems clean. These dressings can be carried into the canal in this manner. The fine platanoid probes as they come to us, have pointed ends, take one pointed at right angles to the oil stone, and draw it a few times across the stone, making it squarely blunt so that it will not so easily pass through the cotton. It is not always necessary, but it helps many times in small canals, to stick the point of probe into wax, just touching it, then engage a trace of cotton fibre on the wax, and twist the probe, and you can easily wind on a very small amount of cotton, which tapers so nicely that it can be carried well into the canal until it reaches a portion of the canal as small as the probe end. Now reverse the probe, and with a tamping motion, the cotton will leave the probe, and can be worked into canal, leaving just enough at orifice to draw it out by. This is a good method also for carrying medicaments into canals.

I usually leave such a dressing in each canal to insure keeping canal clean in case the stopping should come out before patient returns. After the dry dressing has taken up all the moisture, I use either eucalyptol or iodine and phenol in preference to the stronger oils.

If a thin exudate continues, a dry powder of iodoform is sometimes packed in for a day or two. Occasionally these cases will yield with very little treatment, especially in the upper teeth, but more often in the lower teeth, where it is more difficult to raise or drain off the pus, it is necessary to establish better drainage by opening through gum and process at nearest point possible toward apex of root. In doing this cocaine may be used, but the parts do not heal so readily as they would if a little phenol, only is touched to the gum at point where the incision is to be made.

Having established drainage with the knife or bur, or both, wash both canal and fistula with warm, boiled water, or other non-irritating solution. This can be done usually with a strong syringe by plugging around point of syringe with soft rubber.

I use the little rubbers that come with the Dunn syringe, but I use them on a much stronger syringe. Having cleaned the tooth and pocket as thoroughly as possible, it is well to flood the canals and cavity with iodized phenol, and force this through into the pocket by pressure, as you would in pressure anesthesia, repeating it until iodine shows through fiistnla.

A few shreds of cotton with iodized phenol should be left in the canals and also in the fistula to keep it open. The cavity may be plugged tight, and the patient dismissed for from 24 to 48 hours.

When the patient returns, place the dam before removing the stopping, then examine the canal dressings carefully to determine if there is still a trace of pus.

Next, wash well with alcohol and dry with hot air. If there is still pus, or the tooth is sore, repeat the treatment. If quite sure there is no pus, fill the canals, but keep the fistula open another day or two.

If, however, a case is presented that has become chronic, one with a fistula, or one that has a large pocket in the alveolus which has become necrosed, it may be necessary to so enlarge the fistulous tract, either immediately, or by the slower process of packing with compressed sponge sufficiently to permit of cutting away all necrosed bone with a large and sharp bur. In these cases, unless I wish to use the canals for better irrigation, I fill the canals as soon as they are sterile, and finish the treatment through the fistula, keeping it well open at orifice to allow of granulations filling in from the base. Sometimes it is advisable to trim roughened root ends.

If a case is presented in which there is much pain and pus, as soon as the pus and debris has been removed from the cavity, a cresol and formalin preparation may be placed in the cavity and the cavity tightly sealed for from 24 to 48 hours, as suggested by Dr. Buckley.

He says, "In establishing asepsis, we should select and use such remedies as will chemically change the end products (infectious materials) with the least inconvenience to our patients and to ourselves. This can be accomplished by adjusting the rubber dam, sterilizing the teeth included, and then with a large round bur, opening into the pulp chamber, and mechanically removing the debris, but making no attempt to remove the contents of the canals. Now hermetically seal into the pulp chamber on a small pledget of cotton, equal parts of formalin and cresol, seal with a quick-setting cement, thus being certain that the remedy is hermetically sealed, and avoiding pressure. This treatment can remain until you desire to have the patient return for a subsequent sitting. It can be changed the following day, or it can remain a week or more. At the second sitting, the dam being adjusted, the dressing is removed, and the canals thoroughly cleaned and dried with alcohol, after which the remedy can be placed in each canal on cotton, and the cavity again hermetically sealed. It is well to allow this dressing to remain for at least three days, by which time it will have sterilized the entire tubular structure of the dentine, thus establishing asepsis. All that is necessary to prevent renewing sepsis, is to thoroughly fill the canals."

For years we have all experienced trouble, and our patients much pain, from the sealing of cavities too tightly when pus and gas were forming.

Cresole and Formalin act upon the ammonia and hydrogen sulphid gases, thus relieving the gas pressure, and at the same time thoroughly sterilizing the tooth substance. It would seem that this is one of the most rational remedies.

I do not know that much time in the end is saved by the use of this remedy, but if it will stop pain and make the tooth aseptic and enable us to quickly turn off a patient who has come at an inopportune time, it will be a useful practice, especially if its action continues for some days, one need not feel hurried in arranging a subsequent sitting.

I have not used this method in many cases as yet. It has been used in the college clinic with varying success. Often followed by pain, but in these cases the pain may have been due either to the fact that the students, in attempting to clean the canals, have forced septic matter through the apex, or perhaps too much of the preparation was used, causing too much pressure, or perhaps too much pressure may have been exerted in sealing.

Dr. Buckly clearly states that the remedy is not intended for every kind of case presented. And we all know that the personal equation of the operator, in whatever method he uses, has much to do with the result.

For some time past I have been using Alphazone with success, as a disinfectant in place of hydrogen peroxide. Alphazone is an organic peroxide, but, unlike other peroxides, it does not effervesce and cause pain when sealed in a cavity. Although it has been proven to be many times stronger in its germicidal action than phenol, formalin or cresol, it does not irritate the tissues of the mouth. It is particularly useful in cases where it is impossible to place a dam. In these cases, the canals and the whole mouth as well, can be washed thoroughly with the solution. It is slightly acid, and leaves a faintly metallic taste. Most patients do not even speak of the taste. It is not stable after two or there days, but a one one-thousandth solution is available almost as soon as a pellet is dropped in two oz. of water, so it is easily made fresh each day.

In order to be effective, it must come in contact with the pus. As it can be used with a syringe, it is well adapted for washing abscesses and fistulous tracts.

After placing the dam and opening cavity and canals mechanically, as previously described, I wash the cavity and canals several times with the solution, and before dismissing the patient, place a dressing saturated with the dressing in each canal and seal up the cavity.

Within the past two weeks I have had a number of badly broken roots that I have treated in this way. Some I treated three or four times and some only twice before filling the canals. Alphazone is valuable as a deodorizer, and is said to be very effective as a bleaching agent, but I have not had occasion to use it in that way. As it is somewhat acid in its action, the tooth treated should be neutralized with a bicarbonate of soda solution before filling permanently.

Whatever remedy is used in the treatment of teeth, it seems to me the best and most lasting results can only be obtained by the utmost care being taken to so thoroughly open every canal that not only shall debris be removed, but that we shall be able to reach all parts with our medicaments, and also, that we shall be able to hermetically seal the roots from end to end.

DISCUSSION.

DR. OAKMAN. I was very anxious to see this subject brought out at some of the meetings during this year, but the essayist has taken a course I did not anticipate. I thought his paper would include another phase of the subject with regard to instrumentation specially. I have had no opportunity to read the paper until this evening, being out of the city for several days, consequently I am unable to discuss this paper as I would like.

I do not use the essayist's method of treatment. I know very well Dr. Buckley's method of treatment. We can give the credit for most excellent work done by Dr. Buckley in therapeutics as far as the treatment of teeth goes.

The Essayist speaks of using iodine combined with phenol. That is an excellent agent, for the simple reason that the coagulating properties of the phenol are greatly reduced by the iodine and we know that iodine is good for any tissue that is in a pathological condition. Iodine as an alterative acts in a way that you cannot describe, but we do know that the results are very satisfactory. In treating diseases of the mouth of any description iodine has been laid aside by some dentists and other drugs used, far inferior in my estimation.

We cannot properly cleanse root canals by using a larger broach than the canal will admit without great pressure, and if we are able to force the large broach into the canal as the essayist says we force some of the septic substance through the roots. This is also true in the removal of the pulp of a tooth. How often in our earlier practice have we tried to remove the pulp with a broach possibly three times the size of the opening to the canal. We invariably broke off the broach. I guess we can all confess too, that we have sometimes punctured the root of the tooth with our burs which is usually done by using too small a burr when we are cutting down to the floor of the pulp chamber. If we use large burrs the possibility of perforating the floor of the pulp chamber at the bification of the roots are materially lessened.

It has been my practice in treating chronic abscesses, when they do not yield to treatment readily, to use a local anesthetic and amputate the roots of the teeth. I do not mean all three roots of an upper molar if it is not necessary, but where you can amputate one or two roots of these, or one root of an inferior tooth, this treatment will result most satisfactorily in many cases. How often have you filled teeth where you have had chronic abscesses treated for several weeks, and in six weeks the patient returned with a recurrence of the condition worse than before. If you had gone through the process with a sharp burr and satisfied yourself that you have a carious or necroses condition of the bone, where medicine cannot effectually be employed, and surgically treated it you would have a permanent result.

I do not agree with the essayist when he says cocaine prevents the healing of a wound, and instead of cocaine uses phenol as an obtundent. That is not my experience, I am a firm believer in the use of cocaine, providing I have a sterile solution, properly fortified so that you can have no toxic effects from the drug. I think the use of a sterile solution of cocaine injected into the tissues, frequently aids in the healing process.

I dare say that not a man in this room has given up entirely the use of hydrogen peroxide in the root canal treatment. I have stopped the use of it in the last three years. We should not inject into a small fistula hydrogen per oxide full strength, as the effervescence may result in trauematic infection. Many cases of carious bone or necrosis have resulted from injections into blind abscess pockets. The fistula may be at the end of the root of the tooth, but when you inject per oxide into the tooth canal it is not always possible to get it to pass out freely through the external orifice of the fistula. The sooner dentists stop the use of per oxide of hydrogen in root canals, and more especially in those of the lower jaw, the less caries and necrosis there will be to deal with.

It has been my practice to use formalin in a four per cent solution a great deal in the treatment of putrescent canals. I think the treatment that the essayist has recommended is a very excellent one, but it has not been my good fortune to have used it.

I also want to add a word of caution that when you have abscesses in the region of the molars and bicuspids of the lower jaw and the abscesses have persisted for weeks or months, don't fill such roots at once and send the patient away thinking he will be all right. Unless you destroy all the pus, and are sure all the pus is absent, you will sooner or later have alveolar trouble. Yesterday, in Battle Creek, a dentist was having trouble with an upper left first molar, treated twelve years ago. I was able easily to force a steel instrument through the process into the antrum. Injecting cocaine, I was able to amputate the root. Had the amputation of the roots been done years ago it would have saved him his present trouble which may last him many months.

It is a mistaken idea that one can have antrum trouble only from a diseased tooth, or from an abscess about the roots of the first or second molars. I have seen it from the third molar and all the other teeth through to the centrals. I have seen it in many instances, caused only by improper root fillings, or neglected abscesses. There was a time when we dwelt heavily on the use of sulphuric acid in the treatment of putrescent pulps. Treat such a tooth thoroughly with sulphuric acid, and in from six months to two years, the patient may come back with the tooth crown broken. That occurs many times, but few of us realize the cause. The fact of the matter is that sulphuric acid disintegrates the teeth and leaves no life in them. I only use sulphuric acid in the treatment of putrescent pulp when I expect to crown them.

I think another difficulty, especially with the young practitioners is that when they have a putrescent pulp they try to get that tooth well in about two days. If they would only open into the tooth and let out the gas, and put in a little loose cotton without medicine they would often get better results. We have all been guilty of trying to cure the disease too quickly, with the result that we have retarded the process of healing and frequently lost the tooth.

Dr. Ward. I have been using the cresol and formalin formula. that Dr. Buckley speaks of in the Cosmos of 1906. I could not discard it in the treatment of absecesses for anything else.

I disagree with Dr. Oakman particularly in regard to one thing in the use of this formula, that is, the strength of the remedy that should be used. I think the thing we all overlooked in the treatment of teeth is, not the strength of the solution particularly, nor even treatment that we are using, but the effectiviness and thoughness of application. You can place the strongest antiseptic there is made in the tooth and it will be perfectly inert if the substance is not volatile. The main two things in the essayists treatment is that the Cresol and formaldehyde are decidedly volatile. If you have an abscess that you want to take care of right away you would have to cleanse the root canal first and next break up the fistulous tract. Will you do it by surgical or medicinal treatment? I prefer to use some drug that is volatile enough to break up the abscess by sending it into the abscess through the tooth. Very rarely do I find occasion to amputate a root, and rarely find recurrence of that same abscess if it is cured with the Buckley or other proper medicinal treatment. I think the main thing is the volatility and consequent penetration of the treatment rather than its strength. I would explain the things that Dr. Hall speaks of in regard to pain by saying that the treatment must not be pushed through the root canal into the soft tissues for if formaldehyde be pushed through the end of the root it will surely cause pain. Another thing that does not appeal to me is the treatment with alphozone in a solution of water. Now no matter how much good it does when placed in the vicinity of the disease, if you don't get it into actual contact it is not of much value as it is not volatile. It is the same with iodine and phenol, which is not a volatile compound and to use phenol in one of these old chronic cases and expect to cure it immediately, will result in disappointment. The object is to keep the treatment in the pulp chamber, and be sure thet the root is clean and wait for volatile results rather than attempt direct application of strong couterant remedies.

For putrescent pulps where there is pus present the essayist cleanses the canals first and gets drainage, so that he can irrigate the sinus through the tooth canal. He then places in the canal equal parts of formaline and cresol or other antiseptics, but he does not try to get through the canal with the broach at the first treatment or he might push septic matter through into the apical region before he has sterilized the canals. After he has had time to sterilize them then he irrigates the canal and fistulae and gets them into shape so that he can fill them. It is my opinion that you can use irrigation with antiseptics before the pus has stopped forming.

DR. McDONALD. I can hardly agree with Dr. Ward's statement in regard to the volatility of the liquids which he uses for putrescent root canals. He starts in with the assertion that treatments or medicines owe their usefulness really to their volatility. From that he concludes that alphozone or anything else that is not volatile cannot be of any use. Non volatile agents decompose in the presence of pus readily. I use Dr. Hall's method of disinfecting root canals and have used it for several years and so I can hardly agree with that statement. I have used cresol, phenol, and oil of cassia, and have had some very satisfactory results. I believe that root canals are made healthy because I use these medicines, and I believe that there are many in this hall tonight who are using cresole and phenol and are getting good results. I also believe that we can get better results from Dr. Buckley's formula and method than we can with any of the commercial formulae on the market. We cannot use the same strength in all cases, however; I find in the severely distressing conditions you can use 50 per cent solution of formalin and tri cresol and get good results. Supposing you have a case where the putrescent condition is not very severe; or supposing it is a case where there is very little gas forming and yet there is a certain putrescent condition, you can make strong applications of the remedy. I have found that in cases where the putrescent condition was not bad that severe pain followed the use of the formaline solution, but I found that by limiting the quantity of formaline the pain decreased and I got the same result. I believe that where you fail to get good results from these severely depressing conditions it was because you failed to use the full strength solution. Where you can get good drainage I believe that you can get better results by using tri chloracetic acid than anything else. If you have to open into the pus cavity through the process as well as the tooth, I believe you can get just as good results from tri chloracetic acid as from anything else. I use it in place of phenol simply because the escharotic effect is not so deep. There is no doubt that when you use phenol in great quantities you can cause the very condition you are trying to avoid. Of course where a portion of the cementum of the root is affected I believe nothing but the use of sulphuric acid, or the surgical operation, will suffice.

Dr. Ward. I want to correct the impression that it is my opinion that it was entirely the volatility that cured abscesses. I did not mean to convey that impression alone. Dr. McDonald speaks of tri cresol, which is a different reagent entirely from phenol. Tricresol takes care of a wonderful number of abscesses, as every one knows who has tried it, and phenol will do just about the same thing, only not quite so active.

A Member. In order to get the medicament into the fistula of abscesses of the upper teeth, we must have volatility, but gravity takes care of it in the lower. It is vastly different whether it is the upper or lower jaw whether volatility is the all-essential element In regard to this article by Dr. Buckley. As I remember it, it made a great deal of difference in opening these teeth what conditions we find as to whether it is necessary to seal it in immediately or not. Dr. Hall has given the idea that we should first leave the treatment in long enough to get the pulp in as nearly aseptic condition as possible at the first treatment. Some one has mentioned the fact that if there was a copious flow of pus that it would not be wise to seal in such a remedy. I understand Dr. Buckley does that very thing; he washes it out and seals this treatment in and leaves it a little time, and then takes it out and washes the cavity and seals in another treatment of the formula; each time he decreases the amount. But you want to keep the high percentage of the formula as long as you have a copious flow of pus. I have used this in a large number of cases and I have received excellent results, and in all my cases there has been only one case where there was a fistula.

DR. C. H. LAND. Before any of these remedies came into existence I practiced and found in those old times that the man who got to the bottom of the root almost always succeeded, but if you cannot in a mechanical way clean out a root with water and alcohol, or if you are going to depend on some drug that is going to travel up the canal, it makes you treat the tooth for months and are never sure it is well. In the olden times we did not often fill the root canals with gutta percha; we simply washed them out with alcohol and water and then filled them with gold and I know many that have been in there 20 years. In my experience the lower molars, and some of the distal molars are the ones that puzzle us all. If I knew of a remedy that would go up through those canals and I should not have to clean them, I should be glad to get it. We depend entirely too much on the drug idea. I wait a long time before I take chances and fill a root where I have not got to the bottom of it. I lose some, but I have pretty good success. Usually when we are talking of treating and filling roots we should say what tooth. One trouble I have found, if you use formaldehyde too strong you have a very sore root for some time. Alphazone I have used successfully, in several cases, but I used it dry and I use no water at all with it. If you use alphazone dry, where there is bleeding it will stop it.

DR. GRAHAM. There was one thing that impressed me somewhat in the discussion of Dr. Oakman, when he spoke of amputating roots. I think it is not as we do it so formidable as many of us would think. It is not necessary that we actually cut in there and remove part of the root; often is only a certain root that is necrosed, and if there is any ortion of the root diseased, it can be removed very easily with a burr. So far as amputation of the roots is concerned, this is very seldom necessary in my experience, but I do not hesitate if after a few treatments by the latest and most improved methods, and the tooth does not get better, and if I have found that the tooth canal is aseptic enough, I usually fill that root canal, then by means of phenol and cocaine I open up and remove all the necrotic tissue that I find surrounding the apex of that root and the necrotic tissue, being very much softened, will be removed, and the healthy, hard tissue will not yield to the burr. It is often only necessary to remove the necrotic tissue and not any great amount of the roots. However, if you have a larger necrosis followed by disintegration of the tissue you have to remove part of the root, and its removal of course is followed very often by very good results. We have heard that it is necessary to polish these roots very thoroughly. If we take the time to thoroughly open up the field of operation, by sponge, gauze or any other means, so that we can see the field thoroughly then we can know whether it is necessary to amputate or not, and, as I said before, it is not necessary that we should, in many cases, go on and do a thorough operation. I wash it out with a mild antiseptic solution, and I sometimes pack it very lightly with carbolic or any other sterile gauze, and possibly give it another treatment later on. If I have found that the necrotic area is very small I wash it out and put in another packing, until I find that the results justify the procedure. I have found also that instead of gauze, which sometimes is followed by very unpleasant results on account of the gauze packing absorbing, and if the area is quite large, I take a piece of modeling compound that has been boiled and sterilized and roll it up and force it into that excavation, and if it is anterior or posterior I simply press down the lip or cheek and let it remain there. I find that it is a good thing instead of the gauze. That prevents the superficial granulation from the lower areas. If I find that the necrosis is posterior to the roots, of course then it is very often necessary to remove and amputate part of the root, but I think if many of you have used this modeling compound you will find it very valuable used as I have said. The gauze is not very aseptic and it very often will become removed.

Dr. Oakman. Dr. Graham says that after removing a carious area he packs it very lightly and then when the patient comes back the gauze is lost. That root should be packed tightly so you can look into it and keep close observation of that wound for several days and keep track of the whole area. Pack the wound tightly, it is a mistake to pack it loosely. We take for instance a central incisor. Down lingually in that root is carious bone. Now if you pack it lightly the orifice of the wound is not dilated and you do not know whether you have all the carious bone out or not. When you have the parts anesthetized is the time to pack the wound.

Another good way is to pack it with wax which has been well boiled and is sterile, and use in the same way. The gauze should be removed daily and should not be kept there longer than you want to view the field.

Dr. Graham. The doctor misunderstood me. I had special reference to those cases where we had small necrotic areas. In those cases it is very often only necessary to place a small packing of gauze, and it is not necessary to pack it so tightly, because it is not necessary that we treat it again except by irrigation. If, as I said before, the area of necrosis is large and you wish to view the area of the root again it is necessary to have the packing so that it will expand the tissues about it. But in the great majority of cases I will say that when I have found the canals are aseptic I immediately fill. Sometimes I fill them when I find a little pus is oozing out; of course with gutta percha in the apex, it is not necessary to always pack that so completely. I fill the root canals before I get all the pus out of the canal because it saves time, and you can treat from the outside.

Dr. Hall. If I was going to use the drill I would use the largest size first, because that point where the canal leaves the pulp chamber is the largest sized of all roots; however, I do not go down into the root canals with Gates drill so much as I used to. I have broken off some and had some difficulty in getting them out. I use the broaches as put out by Kerr and Downey. I use the largest first and then follow the next size and so on until I get the canal pretty well opened. I believe if we open these canals pretty well you can get a good way into them and pretty close to the apex of most of them. By thoroughly opening the pulp chamber and getting good access to it, and enlarging the orifice to the canal of the pulp chamber, I think you can go actually on further than a good many people do towards the apex of the root, and, having gone as far as possible you can surely get your treatment further on and good canal fillings as well. I do not believe that it makes very much difference whether it is upper or lower teeth as to whether the medicinal agents will travel up or down. If you open your canal as much as possible I think you can carry that material into the canal by capillary attraction by running a very fine broach alongside of the canal, brings out the air, thereby getting your medicinal agent up past the air.

I did not wish Dr. Oakman to understand that I would inject the carbolic acid in the gum in order to make the incision towards the apex of the root; I would simply touch the point to the gum with a very small trace of carbolic acid in order to prevent pain in going through the mucous membrane. After you have prevented that little pin prick going through the mucous membrane it is not so very painful to push the instrument into the sinus.

Now for the amputation of the root. It is only in those cases where the root has become slightly necrosed, as well as the alveolar process. Occasionally you will find the root, when you have pain, very rough and necrosed on the side. In those cases I would burr it off, but it is seldom that I burr off the end of the root, and I do not believe I have had many cases that have gone out for antrum treatment because I have not cut off the end of the root. I know Dr. Oakman sees a great many more antrum cases than I do, but I do not believe he has seen many roots that have been properly treated that should have been cut off.

After filling the root canal it has always been my practice not to fill the tooth permanently immediately afterwards. I do not just know why I do this, because I do not have very mvch after-trouble with the root canal treatment, but I am conservative in it and always put in temporary fillings of gutta percha or something else for several days after I have finally filled the root canal. I think it is highly important when filling the root canal, not to coat them all over with gutta percha but to get just enough in to fill the root canal and then firmly place the gutta percha points. I frequently insert a little eucalyptol first and then put in the gutta percha. If you withdraw the point and twist it when replacing you will let the air get out faster, and the eucalyptol helps to force out the air. It softens the compounds after it has been in there a moment or two, and by the time you have started the point in the canal the second time you can go back to the first canal and push it in a bit further, and in that way you can get the root canals pretty securely filled.

Dr. Ward. I do not believe alphozone will disinfect and therefore can be of no service. I have not used it in just that way with the purpose of disinfection. I am using it in canals where there is no great amount of pus, excepting in those canals where there was an abscess at the end of the root and fistulous opening. I believe in these particular cases it is especially well adapted, because you can force it into the abscess direct with a strong syringe and out through the fistulous opening and feel sure that you have touched every part of it. Now these other drugs which you can only use a small part of, like iodine or formaldehyde, you cannot put into the syringe and force them through. The alphozone does not effervesce as do per oxide of hydrogen and dioxogen.

Dr. Thompson. Dr. Hall says that in treating putrescent pulps if you found vitality in a portion remaining that you could use a little cocaine. I think that is a dangerous practice. If you have any pressure, it is much better to dry up that remaining portion of the pulp.

A Member. I recall an experience with the Illinois Dental Society. One year they sent out to a number of prominent dentisis throughout the state 100 molars imbedded in Plaster of Paris casts to be filled and numbered and returned to the Society to be opened at a meeting. These were all to be filled in the dentists own way, their usual way of filling and treating the roots, and of course under such a test as that they would use all of the care in dentistry that was possible to fill these roots. When they broke the casts and examined the roots, out of the 100 there were only three perfect fillings. I think that shows about what percentage of the roots are perfectly filled. I do not know how the three were filled that were filled perfectly, but one of them was filled as Dr. Hall speaks of, by putting in first the eucalyptol, then the gutta percha, followed by the point. The other 97 were all imperfect fillings.

Dr. Wood. I do not profess to fill more than a thousand of these teeth a year; I heard one gentleman say he filled a thousand of them, and I wondered what kind of a practice he had.

In regard to eucalyptol in filling root canals. I can assure you it has given me a great deal of comfort and satisfaction. It is the most saving thing that we can put into root canals, even though we do sometimes force a little medicine through. It leaves the tooth quiet and it stays quiet. In forcing in the gutta percha point after putting in the eucalyptol I always either apply a hot instrument to that pulp chamber or play hot water over and warm that point and then work it down with a fine point, and I believe I get better results in that way than in trying to force it in the hard condition, depending on the softening of the eucalyptus.

Dr. Graham speaks of filling these roots just as soon as he got them dried out and thoroughly cleansed, whether the pus was stopped or not. Now I do that. I have always done it, simply with the idea that I considered you have got that much trouble stopped, and that much of your field clean and filled and no more pus or trouble can get in there.

DR. C. G. BOWLES. One phase of this subject I would like to hear discussed, is the one that presents sometimes when the swelling has just started outside of the jaw. The patient comes to you with a great deal of pain and you can not open it. Then how are you going to give that patient relief? My experience has been that they have got to suffer 5 or 6 days until an abscess presents. Some one has had perhaps unusual success in that line of cases, and we should like to hear from them.

DR. BUTTRICK. A little dental apparatus made by Cochin, in Charleston, S. C., I believe, will bring the abscess to a head very quickly, and does draw to a head sometimes and gives relief. It consists of a little emulsive poultice, which, when put into boiling water will swell up and you put that in the mouth between the lip and gum.

DR. E. C. MOORE. I have tried taking soft plaster and putting it around the abscessed tooth, so that the plaster is also attached to the other teeth, and I might say making a plaster splint, and after it hardened drill through it to the abscessed tooth, cutting through any filling that might be there, and the absecessed tooth suffers no jar that is sufficient to be painful, and one can get into the root canal of an abscessed tooth that way. I have seen it mentioned in the dental journals to use modeling compound in the same way, but it struck me that plaster would hold it firmer. In my experience I have found it to answer very nicely. The greatest trouble with me seems to be to cut into the tooth at all, but after getting it opened up, go ahead and use the broaches and with the escape of gas relief comes.

DR. STRAITH. While I persisted in the use of the treatment that was taught to me in my first term in college, and while it very seldom failed me, yet I had some obstinate cases that it would not reach and could not be reached in any way that was suggested to me. I remember one case I had in Bay City a patient who never had lateral incissors. The central incisors were separated quite a little with quite a large space between them and the cuspids and those teeth had been filled a number of times with gold, so that they were more nearly gold fillings than tooth substance, and she insisted upon having these teeth extracted. Very much, I presume, to your surprise, I insisted that they should not be extracted, and finally advised the putting on of crowns. I treated those teeth, devitalized them and used the treatment that I had always used and left them for three or four weeks before I would attempt to put crowns on them and they kept sore and finally abscessed. One of these teeth had a fistula. I used the treatment for that that I had always used, that was to wash the mouth with some aseptic solution and follow with a little peroxide, and then full strength carbolic acid and fill the root canal immediately, and a vast majority that I have treated in that way I have never heard from and I think if there had been any trouble they would have come back to me. However, I remember one or two that were not all right. As to my knowledge of these teeth that have been extracted, it sometimes amuses me to hear others tell how successful they are in filling every root canal. I take out teeth occasionally that have roots that are far from straight and I expect if those roots were filled down so as to expose the root canal from the pulp chamber to the apex it would be all gold, and put a gold foil filling in there, or even a very pliable gutta percha point, let alone the idea of putting in a broach and taking out the contents, be it a live pulp or much less be it putrescent pulp. I find many pulp chambers that have been perforated but in most cases where I have found roots fractured I have not censured those who did it very severely because the perforation is almost always below the pulp chamber.

I failed to conclude the story of the lady I saw in Bay City. I refused to extract the teeth, and she finally had them extracted and a bridge put in, and I asked the dentist afterwards if he found anything wrong with the roots and he said he could not tell what was wrong with them no necrosed bone and they looked healthy nor was there anything to indicate trouble.

